# Transcatheter Closure of Patent Ductus Arteriosus: 11 Years of Clinical Experience in Cipto Mangunkusumo Hospital, Jakarta, Indonesia

**DOI:** 10.1007/s00246-015-1128-2

**Published:** 2015-03-07

**Authors:** Mulyadi M. Djer, Dimas Dwi Saputro, Sukman Tulus Putra, Nikmah Salamia Idris

**Affiliations:** Pediatric Cardiology Division, Department of Child Health, Medical School University of Indonesia-Cipto Mangunkusumo Hospital, Jl. Diponegoro 71, Jakarta, Indonesia

**Keywords:** Transcatheter closure, Patent ductus arteriosus, Amplatzer duct occluder, Coil

## Abstract

Transcatheter closure of patent ductus arteriosus (PDA) has been suggested to be the standard treatment of PDA. Although, in general, the procedure shows a high successful rate, outcomes may vary among pediatric cardiology centers. To evaluate the effectiveness of transcatheter closure of PDA in Cipto Mangunkusumo Hospital, Jakarta, Indonesia, this was a retrospective study on patients who underwent transcatheter closure of PDA in Cipto Mangunkusumo Hospital during the period of 2002–2013. Hospital registry was reviewed and data about patients’ characteristics, PDA severity, procedure, and outcomes were retrieved. There were 298 patients, of whom 90 were males, who underwent transcatheter closure of PDA during the study period. Median age was 3.4 years (1 months–18 years), and median body weight was 12 (3.6–59) kg. The diameter of PDA ranged from 1.1 to 15.4 mm with a median of 3.7 mm. Device could be deployed in all patients, in which most were the Amplatzer ductal occluder (69.8 %) and the remainders were coils. Median fluoroscopy time was 15.4 (1.5–87) min, and procedure time was 76 (30–200) min. Complete closure was achieved in most patients (97.3 %), whereas device migration occurred in a minority (0.3 %) of patients. No major complication occurred during or after the procedure. Transient anemia and bradycardia were found in 3.7 and 1.3 % patients, respectively. Most patients were discharged from the hospital at 1 day after the procedure. Transcatheter closure method is a safe and effective procedure to close PDA.

## Introduction

Patent ductus arteriosus (PDA) constitutes 6–11 % of all congenital defects [23]. It is estimated that PDA occurs about 1 in 2500–5000 live births, and there are 4000 infants with PDA born in Indonesia annually [6]. The incidence of PDA is higher in preterm infants, which is about 8 per 1000 babies [6, 23]. PDA, especially the moderate and large ones, often causes heart failures and growth disturbances in children [23].

Transcatheter PDA closure has been routinely performed in the last decade [2, 14, 16, 25, 26]. Since this procedure is less invasive than surgical procedure, it has become the treatment of choice for PDA [7]. Several factors have been suggested to determine the success of PDA closure using these methods, such as the type of PDA anatomy as suggested by Krichenko [15]. In Indonesia, the first PDA transcatheter closure was done almost 16 years ago; however, there has been no previous published study evaluating the outcomes of transcatheter PDA closure among Indonesian children [6]. The aim of this study was to evaluate the outcomes of transcatheter closure of PDA in Cipto Mangunkusumo Hospital, Jakarta, as a national referral hospital in Indonesia.

## Methods

We included patients who underwent transcatheter closure of PDA at Cipto Mangunkusumo Hospital (CMH) from January 2002 until May 2013 and retrospectively analyzed their medical records, echocardiographic findings, hemodynamic data, and early follow-up results. Selection criteria for transcatheter closure of PDA were patients with PDA, who the murmur was heard, had bounding pulse, and their body weight ≥6 kg. All the PDA should be closed except tiny PDA without bounding pulse and murmur. Surgical ligation of PDA was indicated in neonates, tubular type of PDA >5.5 mm or large PDA >12 mm.

The transcatheter closures of PDA were performed at the catheterization laboratory using fluoroscopy guidance. All patients underwent the procedure under general anesthesia. After performing a hemodynamic study, we placed the ductal occluder (Amplatzer ductal occluder-ADO) using a Mullin delivery sheath, a delivery cable, and a loading catheter. After the device was attached by screwing it to the tip of the delivery cable, it was then inserted through the femoral vein under the Mullin sheath to close the PDA. The device was then released from the delivery cable after we confirmed that it was placed correctly, resulting in no or smoky residual shunt on angiography. On the other hand to deliver the coil, we used a 4F multipurpose catheter and we cut the end of catheter to throw out a side hole. The catheter was then inserted until its tip reaching the ascending aorta. A pusher wire to open 1.5 or 2 loops of coil at descending aorta was pushed. All systems were pulled until opened coils entering the ampulla of PDA. The pusher wire and catheter were then push and pull step-by-step up to open the proximal part of coil. Finally, the part of coils was deployed at pulmonary artery at 0.5 or 1 loop.

We obtained information regarding patient clinical characteristics such as age, sex, and weight. The cardiac catheterization data were also retrieved, including the size of the PDA measured as the narrow diameter near the branching point from the descending aorta seen in lateral-view angiogram, type of PDA device, and fluoroscopy time. Type of complications recorded included immediate events, such as failure of device implantation, device embolization, residual shunt, significant obstruction of aortic arch and left pulmonary artery, infective endocarditis, and massive blood loss. According to WHO 2011, anemia in children of 6 months to 5 years was defined when the hemoglobin level reached <11 g/dl. In children >5–11 years was that when the hemoglobin level reached <12 g/dl, for 12–14 years if <12 g/dl, and for boys >15 years if <13 g/dl, and for girls >15 years if <12 g/dl. All patients were then followed up for any evidence of cardiac murmur. Follow-up echocardiography was performed using Philips^®^ Sonos 4500 machine to evaluate residual PDA, left pulmonary artery stenosis, and descending aorta stenosis.

Clinical characteristics, type of occluder device, and immediate results/complications were described as mean, median, or proportion as appropriate. Statistical analyses were performed using SPSS statistics 17.0 for Windows.

## Results

A total of 298 patients who underwent the transcatheter closure procedure between 2002 and May 2013 were enrolled. The baseline characteristics are described in Table 1. There were more females than males with wide ranges of age, body weight, as well as PDA diameter. Successful device deployment was achieved in all patients. Most subjects have their PDA occluded using the Amplatzer device (Table 1).

**Table 1:** Characteristics of the patients (*n* = 298)

Variable	*n* = 298	%
Sex		
Male	90	30.2
Female	208	69.8
Age (range)	3.4 years (1 month–18 years)	
Body weight, median (range), kg	12 (3.6–59)	
Diameter of the duct, median (range), mm	3.7 (1.1–15.4)	
Type of device used		
ADO I	282	94.6
Gianturco coil	16	5.4

*ADO* Amplatzer duct occluder

Outcomes of catheterization and intervention are depicted in Table 2. The procedure time varied widely with a median of approximately 1 h. Sizes of the Gianturco coils were 5 cm × 5 mm and 5 cm × 8 mm. Sizes of ADO I ranged from 4 to 14 mm with the median of 6 mm. Most (97 %) patients achieved complete closure and only one who had significant residual shunt. Device migration was found in one patient with a very large PDA (15.4 mm) who weighed only 11 kg. We initially attempted to close the PDA by a 12-size device.

**Table 2:** Outcomes of catheterization and intervention (*n* = 298)

Variable	Number	%
Procedure time, median (range), min	76 (30–200)	
Fluoroscopy time, median (range), min	15.4 (1.5–87)	
Immediate results		
Complete closure	290	97.3
Small residual shunt	6	2
Moderate residual shunt	1	0.3
Device migration	1	0.3
Complications		
None	283	95
Anemia	11	3.7
Bradycardia	4	1.3
Hospital stay, median (range) days	1 (1–5)	

Among our subjects, we did not find any complication related to vascular access, hemolysis, and massive blood loss (Table 2). Only minority of patients experienced anemia (3.7 %) or transient bradycardia (1.3 %). No death occurred in this study.

## Discussion

In this study, we reported the largest case series of transcatheter closure of PDA in Indonesia. The number of females was more than males, which is similar to findings previously reported in the literature [23]. However, the transcatheter technique to close PDA is not different between genders. Overall, we found that PDA transcatheter closure has a high success rate and rarely causes major complication in most of the cases.

The first transcatheter closure of PDA was performed by Portsman et al. [20] in late 1960s. This initial work was continued by Rashkind et al. [21] in late 1970s, after which it is performed and developed worldwide [2, 14, 16, 25, 26]. When a diagnosis of PDA is established, closure of PDA is recommended, either by surgery [12] or transcatheter closure [2, 14, 16, 25, 26], to avoid pulmonary overflow and prevent infective endocarditis.

Previous studies have shown that transcatheter closure of PDA is quite an established technique with no reported mortality and low morbidity. A retrospective case series of 1808 patients undergoing transcatheter closure of PDA showed that overall PDA closure rate was 94 %, and the rate of major average events were 1.5 % [11]. Only few minor complications were found. The evidence of effectivity and safety of transcatheter PDA closure has been shown in another studies as well [2, 14]. Despite our short follow-up, we also found that the transcatheter closure of PDA is effective and save. There were minimal intraprocedure complications, which were anemia and transient bradycardia.

Before the era of transcatheter method, PDA was ligated surgically. Gross and Hubbard [12] performed the first successful ligation of PDA in late 1930s. Since then, ligation of PDA has been the treatment of choice for PDA closures until transcatheter procedures were introduced. Recently, PDA ligation reserved only for neonatal PDA that does not respond to medical treatment or has a diameter more than 14 mm [6]. In our study, the youngest patient aged 1 month with a body weight of 3.6 kg. Actually, this patient previously had undergone surgical ligation but had residual shunt. Considering the small size of his residual PDA, we decided to close it transcatheterly using a Gianturco coil, which is relatively cheaper compared to other devices. Our policy is to close the PDA using a coil if the diameter is <3 mm, otherwise it is closed using a ductal occluder [6]. In our series, we mostly used ADO and only used coil in approximately 5 % of patients. According to the literature [2, 14, 16, 25, 26], there are some advantages of using ADO over coil. First, the shape of ADO is more appropriate to close PDA in order to avoid residual shunt. Second, the ADO device can be retrieved in case the device does not fit the PDA. Besides that, the ADO device is also less likely to embolize than the coil.

All the PDAs in this series were closed using ADO I and Gianturco coils. Gianturco coils were used to close PDA <3 mm. We used transvenous coil closure approach in all 16 cases. Coils were also used for closing residual surgical ligation of PDA. In these residual PDA ligation, we could not use ADO I because the delivery catheter and sheath could not pass the residual PDA ligation.

We used ADO II to close tubular PDA <5.5 mm because the readily available ADO II could only be used to close PDA up to 5.5 mm, and in this series, we did not have the case. So far, we used ADO II to close small perimembranous VSD, small muscular VSD, and doubly committed sub-arterial (DCSA) VSD. Closing these DCSA VSD using ADO II had potential advantage since it was soft and flexible and did not interfere aortic valve activity. In addition, additional size (AS) of ADO II was not available in our institution. According to the readily available recommendation for closing PDA using ADO I, the minimal body weight is 6 kg. However, in our experience, we successfully closed PDA in patients <6 kg.

In our study, the majority of patients achieved complete closure, and the rate of residual significant shunt was only <1 %. These results were comparable to other previous studies [13, 16]. Due to this small number of subjects with residual shunt, we were not able to statistically evaluate factors related to unsuccessful procedure. In most cases, we only found a smoky residual shunt using coils or ADO I. In one case, we found a small residual shunt that finally disappeared in a 6-month period of follow-up.

We experienced aortic protrusion of the device, that is a possible complication using ADO I in small children, but it did not influence the pressure gradient between ascending and descending aorta. In addition, there was one case of embolization of device to the right pulmonary artery; it was not easy to catch that device. This might because the screwed pin of ADO I was inside the device. We used two snares to catch the device. First snare was used to snare the body of device so the screwed pin protruded out and the second snare then easily captured the device.

In order to monitor heart failure and therapy, some studies reported that B-type natriuretic peptide (BNP) hormone may be useful [1, 4, 5, 9, 10, 17, 18]. It has been recognized that BNP level is higher in children with PDA. This hormone is released by myocyte cells as a response to left atrial and left ventricular dilatation. PDA causes a proportion of blood flowing from the aorta to the pulmonary artery, which leads to excessive blood flow in the pulmonary circulation [6, 23]. This excessive blood flow then drains into the left atrium and left ventricle leading chamber dilatation and left heart failure. In our previous study [9], we found that after PDA closure, the mean BNP level significantly decreased from 58 to 28 pg/ml (*p* = 0.001), which was more prominent in the first month after PDA closure. In that study, which involved 23 children, seven of nine children with a high BNP level before the PDA closure showed a normal BNP level at 6-month follow-up. However, in this study, we did not evaluate the BNP level in our subjects since this examination was not conducted routinely in our setting. The transcatheter closure of PDA can be done using either Amplatzer duct occluder (ADO I) or Gianturco coil. The ADO is made from an alloy called Nitinol wire mesh, containing 55 % nickel and 45 % titanium. There is still a controversy about the effects of nickel contained in this device. Concerns arise since this alloy can lead to intoxication through blood stream and can induce cancer [24]. Ries et al. [22] reported that nickel contained in the device used in the transcatheter closure of atrial septal defect can be released to the blood stream and lead to significant elevation of blood nickel level compared to the level before the procedure. In our previous study [8], we found that the blood nickel level observed at six month after the ADO procedures did not increased significantly, and the concerns about nickel released to the blood stream were not proven. This finding was consistent with previous findings of Coe et al. [3]. In this study, we also did not measure the blood nickel level since it was not performed routinely in our procedure.

Important factors determining the success of a procedure are vascular accessibility, availability of devices, morphology of ductus, and imaging modality [26]. Vascular accessibility is important as thrombosis may be formed in femoral vessels due to the breakage of the endothel. The morphology of the ductus and measurements of its size should be done before the procedure to minimize procedural risks and to select appropriate device. In these procedures, we used femoral artery and vein to insert the sheath. We did not find any thrombosis in our series.

Imaging modality is also an important factor, especially in small children, where portable imaging modality may be beneficial in performing bedside procedure [19]. There are many advantages of transcatheter closure of PDA compared to surgical ligation procedures [7]. The procedure is less invasive, leaving no surgical scar and results in shorter hospitalization as well as lower cost. In general, the length of hospital stay in our study is 1 day as reported in other studies. The success rate is also comparable to surgical ligation [7]. However, the surgical ligation is still indicated in large PDA [6].

In conclusion, transcatheter closure of PDA is a safe and effective method to close PDA in children.
